# Willingness to act in a cardiac arrest and awareness of automated external defibrillators: a survey of the Swedish population

**DOI:** 10.1016/j.resplu.2026.101428

**Published:** 2026-07-27

**Authors:** Therese Djärv, David Fredman, Anders Åkesson, Stefan Jutterdal

**Affiliations:** a Swedish Resuscitation Council, Department of Medicine Solna, Karolinska Institutet and Department of Emergency Medicine, Karolinska University Hospital, Sweden; b Heartrunner Sweden AB, Stockholm, Sweden; c The Swedish Heart and Lung Association, Stockholm, Sweden; d Hjartuppropet, Survivor of a Cardiac Arrest, Kalmar, Sweden

**Keywords:** Awareness, General population, AED

## Abstract

**Purpose:**

A population-based survey on opinions about CPR education, willingness to act, and awareness of AEDs location.

**Method:**

A survey was compiled by the commercial Novus Group International, Stockholm, Sweden in January 2026. Novus use their *Sweden Panel*, which aim to mirror the public opinion and consist of about 60,000 people aged 18–84 years. Novus uses purposeful recruitment of representative participants until the target 1000 people have replied to the survey.

**Results:**

Among the 1026 responders, there was an equal distribution of age, gender and one third reported training CPR within 24 months. The majority (*n* = 820,80%) stated that they would be willing to act if an unknown person gets a cardiac arrest, while 7% (*n* = 69) stated that would not be willing to act. Those with recent CPR training were more prone to act and contradictory, those who had never done CPR training (12% of the participants) were most unsure if they would be willing to act. Around half (*n* = 539) feel confident using an AED. Those with a lower degree of comfort was older women (57%), retired (52%) and never have taken CPR training (81%). Half (*n* = 538) know where the closest AED is at their workplace compared to one fifth when they are at home. The majority (*n* = 870) believe it should be mandatory to have government AEDs available 24/7.

**Conclusions:**

A representative sample of the Swedish population is trained in CPR, propose that they are willing to act in a cardiac arrest, but few know where their nearest AED is located when at home. Recent CPR training adds confidence and awareness, but any training seems to have a lifelong effect.

## Graphical abstract

**Figure:**
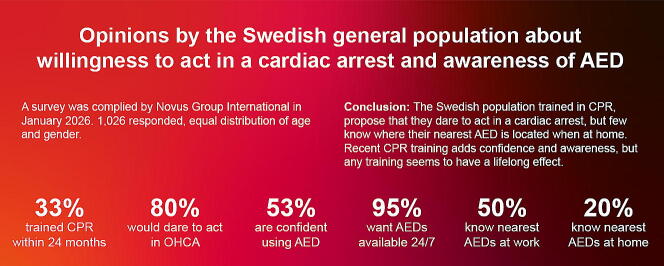

## Introduction

In Sweden 67% of all out-of-hospital cardiac arrest (OHCA) gets bystander-CPR.^1^ Furthermore, in the Swedish AED-registry about 25,000 AEDs are registered, but only one third is available after office hours^2^ and only 8% of OHCA have an AED attached before EMS arrival.^3^ This mismatch might be because 70% of the OHCA occurs at home while most AEDs are in offices.^2^ In recent years the European Resuscitation Council have increased their work on advocacy and awareness campaigns in society. Yet voices from the general population are rare. Therefore we (four organizations; Swedish Resuscitation Council, Heart Runner Sweden AB, The Swedish Heart and Lung Association and Hjartuppropet) aimed to do a population-based survey on opinions about CPR education, willingness to act, and awareness of AEDs location.

## Method

A survey was distributed between 8 and 14 January 2026 to the Sweden Panel by Novus Group International, a public company conducting surveys in society in 50 countries.^4^ The Sweden Panel aim to mirror the public opinion and consist of about 60,000 people aged 18–84 years. Novus Sweden Panel use a what they call a simple random sampling method to recruit, which means that the recruitment initiative always comes from Novus and it is not possible to self-register or “apply” to join the web panel. The random sample is generated based on random selection of phone numbers. Novus perform quality checks regarding time spent per question as well as alignment on questions, further they control so a single person does not participate in too many surveys at one time and not more than 12 per year. Participants get credits for participation; the credits can be transformed to donations to the Swedish Red Cross or a cinema ticket. The survey is sent by e-mail. Novus sends one reminder and use purposeful recruitment of representative participants until the target 1000 people have replied to the survey. Participants have the right to withdraw or change their answers for up to 30 days. Novus adheres to Esomar guidelines, which establish the global ethical and professional standards for market, opinion, and social research, and data analytics.^5^ Novus performs all the data gathering, data analysis and packeting of results. Novus uses 95% confidence interval as standard, and by using a sample of 1000 people the error margin at 5/ significance level is ±2.5% for a distribution of answers 80/20 and ±3.1% for a distribution of 50/50. Characteristics of responders were categorized as in Table 1 and differences between the total sample, and these categories were presented only if classified as statistically significant, i.e. a *p*-value < 0.05.

**Table 1:** Participants in a representative sample survey in January 2026 in Swedish general population about willingness to act in a cardiac arrest and awareness of AED in the society.

**Characteristics, subgroups**	**Number (%)** **1026 (100)**
**Gender**	
Women	509 (50)

**Age category**	
18–34 years	278 (27)
35–49 years	259 (25)
50–64 years	246 (24)
65–84 years	243 (24)

**Type of city**	
Bigger city	359 (35)
Close to big city	377 (37)
Town	290 (28)

**Highest education**	
Gymnasium	601 (59)
University	424 (41)

**Occupation**	
Student	130 (13)
Worker	260 (25)
Public official	273 (27)
Retired	230 (22)

**Civil state**	
Married/Partnership	440 (43)
Living together	187 (18)
Others	395 (38)

**Type of house**	
Rental apartment	265 (26)
Tenant-owned apartment	213 (21)
Own house/townhouse	505 (49)

**Yearly household income**	
Lowest-299 k Swedish Krona (app 30,000 Euro)	175 (17)
Lower intermediate 300–499 k Swedish Krona	198 (19)
Higher intermediate 500–799 k Swedish Krona	224 (22)
Highest (800 k-Swedish Krona)	334 (33)

**Taken CPR course**	
Within 12 months	148 (14)
Within 13–24 months	139 (14)
Within 25–36 months	95 (9)
More than 3 years ago	488 (48)
Never	124 (12)
Not sure	32 (3)

## Results

In total 1026 replied to the survey. There was an equal distribution of gender and age categories (Table 1). About one third (*n* = 288) reported that they trained CPR within 24 months and about half (*n* = 488) stated that they participated in CPR training more than 3 years ago. Only 12% (*n* = 124) stated that they have never participated in a CPR training. The following subgroups responded statistically significantly more often than the total group that they had never undertaken a CPR training, i.e. age 65–84 years (17%), the retired (18%) and the lowest income category, i.e 0–299 k Swedish Krona (app 30,000 Euro) (22%).

**Fig. 1:**
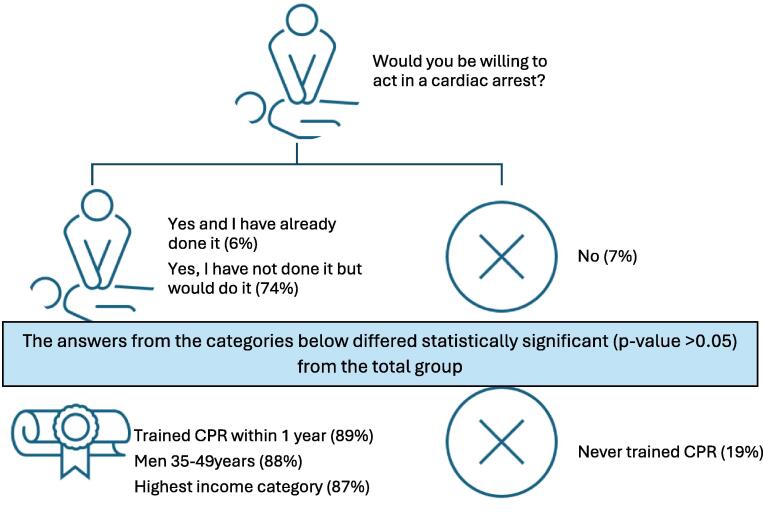
**Hand on heart (common saying in Swedish meaning-to be honest) – Would you be willing to act if someone you don't know suffered a sudden cardiac arrest**? 1026 participants in a representative sample survey in January 2026 in Swedish general population. Difference between the total group and subgroups was interpreted as statistically significant if *p*-value < 0.05.

The vast majority (80%) stated that they would be willing to act if an unknown person got a cardiac arrest (Fig. 1). Those who had taken CPR training within 12 months responded statistically significantly more often that they were willing to act (89%). In contrast, those who had never done CPR training responded statistically significant more often that they were not willing to act (19% compared to 7% of the total sample, *p* < 0.05). Further, women aged 65–84 years reported statistically significantly more often uncertainty in their willingness to act (57% compared to 47% of the total sample).

Around half (53%) feel confident using an AED (Table 2). Participants with statistically significant higher degree of feeling comfortable with using an AED were men (58%), age 35–49 years, employed (63%), those within the highest income category (800 K- Swedish Krona) (65%), those who had CPR training within 12 months (85%) and those within 13–24 months (74%). In contrast, those reporting a lower degree of comfort using an AED than the total sample were characterized by being retired (52%) and never have taken CPR training (81%).

**Table 2:** Questions and answers in a survey to a representative sample of Swedish general population in January 2026 about willingness to act in a cardiac arrest and awareness of AED in the society.

**Question 1: When was the last time you trained CPR?**					
**Within 12 months**	**13**–**24 months ago**	**25**–**36 months ago**	**More than 3 years ago**	**Have never taken one**	**Not sure, can’t remember**
14% (148)	14% (139)	9% (95)	48% (499)	12% (124)	3% (31)
Answers from subgroups differing statistically significantly (*p*-value < 0.05) from the total group*					
Men 50–64 yrs (26%) Worker (23%) Women 35–49 yrs (21%)	Child still living at home (22%) Highest income category (20%)		Retired (59%) 65–84 yrs (56%)	Lowest income category (22%) Retired (18%) 65–84 yrs (17%)	

**Question 2: If you were to find yourself in a situation where you would need to intervene in some way, how safe or unsafe do you think you would feel to use an AED?**					

**Very confident**	**Quite confident**	**Quite unsecure**	**Very unsecure**		**Do not know**

12% (127)	40% (411)	29% (298)	16% (167)		2% (21)
Answers from subgroups differing statistically significantly (*p*-value < 0.05) from the total group*					
CPR training within 12 months (85%) CPR training 13-24montsh ago (74%) Highest income (65%)		Never taken CPR training (80%) Lowest income category (61%) Living in bigger cities (53%)			

**Question 3: If you were to find yourself in a situation where you would need to intervene in some way, how safe or unsafe do you think you would feel to perform CPR?**					

**Very confident**	**Quite confident**	**Quite insecure**	**Very unsecure**		**Do not know**

10% (106)	41% (425)	30% (306)	17% (173)		2% (16)
Answers from subgroups differing statistically significantly (*p*-value < 0.05) from the total group*					
CPR training within 12 months (82%) Worker (62%)		Never taken CPR training (81%) Retired (53%) CPR training more than 3 years ago (53%)			
Preamble *Every year 200*–*250 people die in traffic and since 1997 Sweden has had a zero vision for traffic fatalities. Approximately 90 people die in fires every year in Sweden and since 1999 it has been a law that all newly built homes must have smoke alarms.* *Every year, just over 6000 people suffer cardiac arrest outside of hospital and almost 90% die. Many more could be saved with rapid cardiopulmonary resuscitation (CPR) and the use of defibrillators. Despite this, there are no laws and guidelines in Sweden for CPR training and the placement and availability of defibrillators. There are often several defibrillators in office buildings and shopping centers, but they are inaccessible after closing. Residential areas may lack defibrillators altogether or have them locked away, even though 71% of out-of-hospital cardiac arrests occur in homes.*					

**Question 4: If/when you are considering which party to vote for in the 2026 election, how important are the issues to you, that everyone should know CPR or that defibrillators are available?**					

**Very important**	**Relatively important**	**Relatively unimportant**	**Very unimportant**	**Don’t plan to vote 2026**	**Not sure**

13% (131)	32% (327)	34% (348)	12% (127)	1% (8)	8% (86)
Answers from subgroups differing statistically significantly (*p*-value < 0.05) from the total group*					
Women 65–84 yrs (75%) 65–84 yrs (71%) Retired (70%)		18–34 yrs (60%) Highest income category (60%) Men (53%)			

* Up to three subgroups are presented in falling order.

**Fig. 2:**
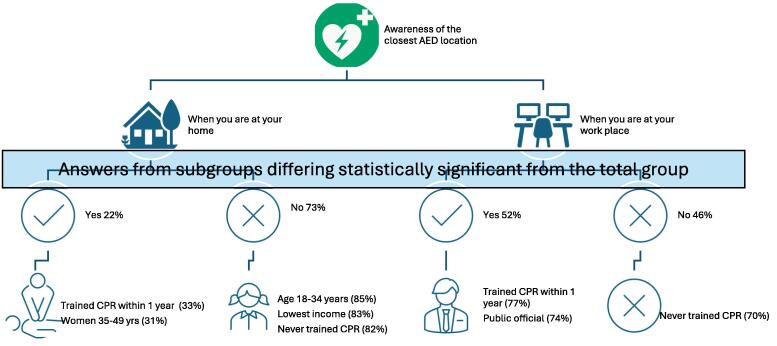
**Do you know where the nearest AED is placed if you are (a) at your work/educational or similar place and (b) at your own home**? 1026 participants in a representative sample survey in January 2026 in Swedish general population. Difference between the total group and subgroups was interpreted as statistically significant if *p*-value < 0.05.

About half (*n* = 538) know where the closest AED is at their workplace but only one fifth (*n* = 224) knows where the nearest AED is when at their home (Fig. 2). Those knowing where the nearest AED at work were characterized by being men 35–49 years, public officials, working in the public sector and have taken CPR training within 12 months. Those knowing where the nearest AED at home where statistically significantly more often women (31% for women aged 35–49 years and 29% for women aged 65–84 years), had an income of 500–799 Swedish Krona (30%) and had CPR training within 12 months (33%). Among all participants, 6% stated that they had their own AED at home. Participants were given a preamble (Table 2) stating that about 25,000 AED are registered in the Swedish AED-registry but only 32% are available 24/7 and then asked how important the 24/7 availability is to them, 63% stated very important and 32% highly important (Table 2). The vast majority believe it should be mandatory to have governmental AEDs available 24/7.

Finally, a preamble about the number of out-of-hospital cardiac arrest in relation to victims due to traffic and fires was shown and participants were then asked how important the issues of everyone knowing CPR and available AEDs are in the Swedish political election in September 2026. In all, 45% stated it as an important issue (Table 2).

## Discussion

This is the first ever survey in a representative sample of the Swedish general population reflecting their voice regarding willingness to act, history of CPR training and knowledge on AED availability in the society. The main messages from the population are that they are willing to act in a cardiac arrest in an unknown person, most have participated in CPR training at some timepoint in their life and in line with previous studies,^6^, ^7^ recent CPR training correlates to the willingness to perform CPR and use an AED. Further, the willingness to act and awareness of the nearest AED seems to be fading with time from training yet yield somewhat of a lifelong awareness and willingness to act.

The method used, i.e. a Novus survey is very frequent used in Sweden for understanding the opinions of the general population, Novus method of random sampling and weighting the answers generate a representative sample of the population. The fact that 67% of all out-of-hospital cardiac arrests in Sweden^3^ currently gets bystander-CPR align with the voice of the population within this survey.

The subgroup of participants reporting no prior CPR training appears to share several characteristics, notably those aged 65–84 years, women and retirement. We speculate that this reflects reduced exposure to routine training opportunities, such as those provided in the workplace or through social networks (e.g. sports clubs). Given that the median age at OHCA in Sweden is 71 years,^1^ and that most events occur in men at home, this subgroup may in fact represent those at greatest risk of witnessing a cardiac arrest and needing to intervene to save a life.

The location of AEDs is currently gathered in a national registry held by the Swedish Resuscitation Council, since the start it has been on an own webpage. However, since April 2026 it is also found in the national 112 app^8^ providing both event alerts and given the exact position when calling the dispatcher center in an emergency.

The current study has several limitations, firstly it was generated as a part of an awareness campaign against media and politicians in Sweden rather than for strict research purposes, the results should be interpreted within that context and the fact that it is a sample of the population. The use of a preamble comparing the number of cardiac arrest victims with those from traffic accidents and fires may have influenced responses. In Sweden, most individuals are accustomed to the widespread use of domestic fire alarms, and many are familiar with the Swedish Transport Administrations *Vision Zero* (*i.e. no one should die or be seriously injured in traffic)* approach to road safety, irrespective of whether they know the exact number of fatalities. This contextual framing may therefore have contributed to a more positive response pattern.

## Conclusion

Within this random representative sample survey, main findings was that the Swedish population is trained in CPR, propose that they are willing to act in case of a cardiac arrest, but few know where their nearest AED is located when at home. Participants who had recent CPR training felt more secure and aware of AED locations, but any training seems to have a lifelong effect since the minority who had never trained CPR often stated that they would not be willing to act and did not know where the nearest AED is located.

## AI statement

AI has not been used in any stage of design, data collection, analysis or presentation including writing up the manuscript.

## CRediT authorship contribution statement

**Therese Djärv:** Writing – review & editing, Writing – original draft, Supervision, Project administration, Methodology, Formal analysis, Data curation, Conceptualization. **David Fredman:** Writing – review & editing, Methodology, Formal analysis, Conceptualization. **Anders Åkesson:** Writing – review & editing, Methodology, Conceptualization. **Stefan Jutterdal:** Writing – review & editing, Project administration, Methodology, Conceptualization.

## Declaration of competing interest

**TD:** Editor in Chief at Visual Journal of Emergency Medicine, Editor Resuscitation Plus.

**DF:** None.

**AA:** None.

**SJ:** “Hjartuppropet” is funded by the companies; HLR Konsulten Sverige AB, HLR Proffsen i Sverige AB, Eldupphör AB and Plusab Medical Solutions AB.
